# Identification of bladder cancer prognostic biomarkers using an ageing gene-related competitive endogenous RNA network

**DOI:** 10.18632/oncotarget.22905

**Published:** 2017-12-04

**Authors:** Changliang Wang, Liang Chen, Yang Yang, Menglei Zhang, Garry Wong

**Affiliations:** ^1^ Faculty of Health Sciences, University of Macau, Macau, China

**Keywords:** ageing gene, bladder cancer, microRNA, competitive endogenous RNA, prognostic biomarker

## Abstract

Competitive endogenous RNAs (ceRNAs) are a newly proposed RNA interaction mechanism that has been associated with the initiation and progression of various cancers. In this study, we constructed an ageing gene related ceRNA network (AgeingCeNet) in bladder cancer. Network analysis revealed that ageing gene ceRNAs have a larger degree and closeness centrality than ageing genes themselves. Notably, the difference of betweenness centrality of ageing genes and their ceRNAs is not significant, suggesting that the ceRNAs of ageing genes and ageing genes themselves both play important communication roles in AgeingCeNet. KEGG pathway enrichment analysis for genes in AgeingCeNet revealed that AgeingCeNet genes are enriched in cancer pathways and several cancer related singaling pathways. We also identified 37 core modules from AgeingCeNet using CFinder software. Next, we identified 2 potential prognostic modules, named K11M14 and K13M4, whose prognostic ability is better than that of age and gender. Finally, we identified microRNAs (miRNAs) regulating the two modules, which include miR-15b-5p, miR-195-5p, miR-30 family members, and several other cancer-related miRNAs. Our study demonstrated that constructing an ageing gene related ceRNA network is a feasible strategy to explore the mechanism of initiation and progression of bladder cancer, which might benefit the treatment of this disease.

## INTRODUCTION

Bladder cancer is the highest incident cancer in urinary system tumours followed by kidney in the United States, with 79,030 new cases projected to be diagnosed (60,490 in male and 18,540 in female) and 16,870 deaths (12,240 in male and 4,630 in female) in 2017 [1]. Currently, the main treatment for bladder cancer is surgery. Unfortunately, high recurrence is one characteristic of bladder cancer [2, 3]. Therefore, it is urgent to identify novel therapeutic targets to improve the diagnosis, prognostic prediction, and ultimately survival outcomes in bladder cancer.

Age is one of the most important risk factors for cancer [4, 5]. Risk for cancer increases significantly after 50 years of age, and half of all cancers occur at 66 years and above. The precise molecular mechanisms by which older people are at higher risk for cancer are an active area of investigation. One current theory posits that as aging occurs, mutations accumulate and long-term chronic inflammation persists, cancer-promoting DNA mutations increase and DNA-damage repair mechanisms weaken. Eventually, a compromised immune and repair system can no longer cope with long term exposure to carcinogens such as sunlight, radiation and environmental chemicals leading to onset of cancer [6]. Nonetheless, a complete understanding of the link between ageing and cancer remains poorly understood. Our study therefore focuses on novel bladder cancer prognostic biomarkers from the perspective of ageing.

MicroRNA is an abundant class of 21-22 nucleotide non-coding RNA that negatively regulates gene expression by inhibiting messenger RNA (mRNA) translation or affecting its stability [7]. The seed region, defined by nucleotides 2-8 of the 5’ portion of the mature miRNA, is crucial for mRNA recognition and silencing. Notably, the interaction between the seed region and mRNA is not unidirectional. mRNA, long noncoding RNA (lncRNA) and other RNA molecules can compete for miRNA to inhibit its activity or to regulated RNAs within their own class. These competitive endogenous RNAs (ceRNAs) act as molecular sponges for a miRNA through their miRNA binding sites (also known as miRNA response elements, MRE), thereby de-repressing target genes of the respective miRNA family. Several studies have verified that PTEN, a tumor suppressor gene, can be regulated by ceRNAs in prostate cancer, glioblastoma and melanoma, via cell proliferation and cancer-related signaling pathways [8]. Our study aims to explore ageing gene related ceRNA modules that have significant prognostic function for bladder cancer patients.

In the present study, we constructed an ageing gene related ceRNA network (AgeingCeNet) specific to bladder cancer. Bladder cancer related mRNA and long non-coding RNA (lncRNA) expression data were obtained from The Cancer Genome Atlas (TCGA) [9] and The Atlas of non-coding RNA in Cancer (TANRIC) [10]. A bladder cancer-specific ceRNA network composed of mRNA and lncRNA was constructed using mRNA/lncRNA expression data, along with experimentally validated miRNA-mRNA and miRNA-lncRNA interaction data. We then constructed an ageing gene related ceRNA network (AgeingCeNet). Network analysis revealed that ageing gene ceRNAs have a larger degree and closeness centrality than aging genes themselves. However, the difference between the betweenness centrality of ageing genes and their ceRNAs is not significant, suggesting that ageing genes and their ceRNAs both play important communication roles in AgeingCeNet. KEGG pathway enrichment analysis for genes in AgeingCeNet revealed that AgeingCeNet genes are enriched in cancer pathways and several cancers related by signaling pathways. Based on CFinder and survival analysis, we obtained two potential prognostic modules. Our results suggest that constructing an ageing gene related ceRNA network could be a novel strategy to identify bladder cancer related prognostic biomarkers.

## RESULTS

### Topological properties analysis and functional enrichment analysis of AgeingCeNet in bladder cancer

**Figure 1:**
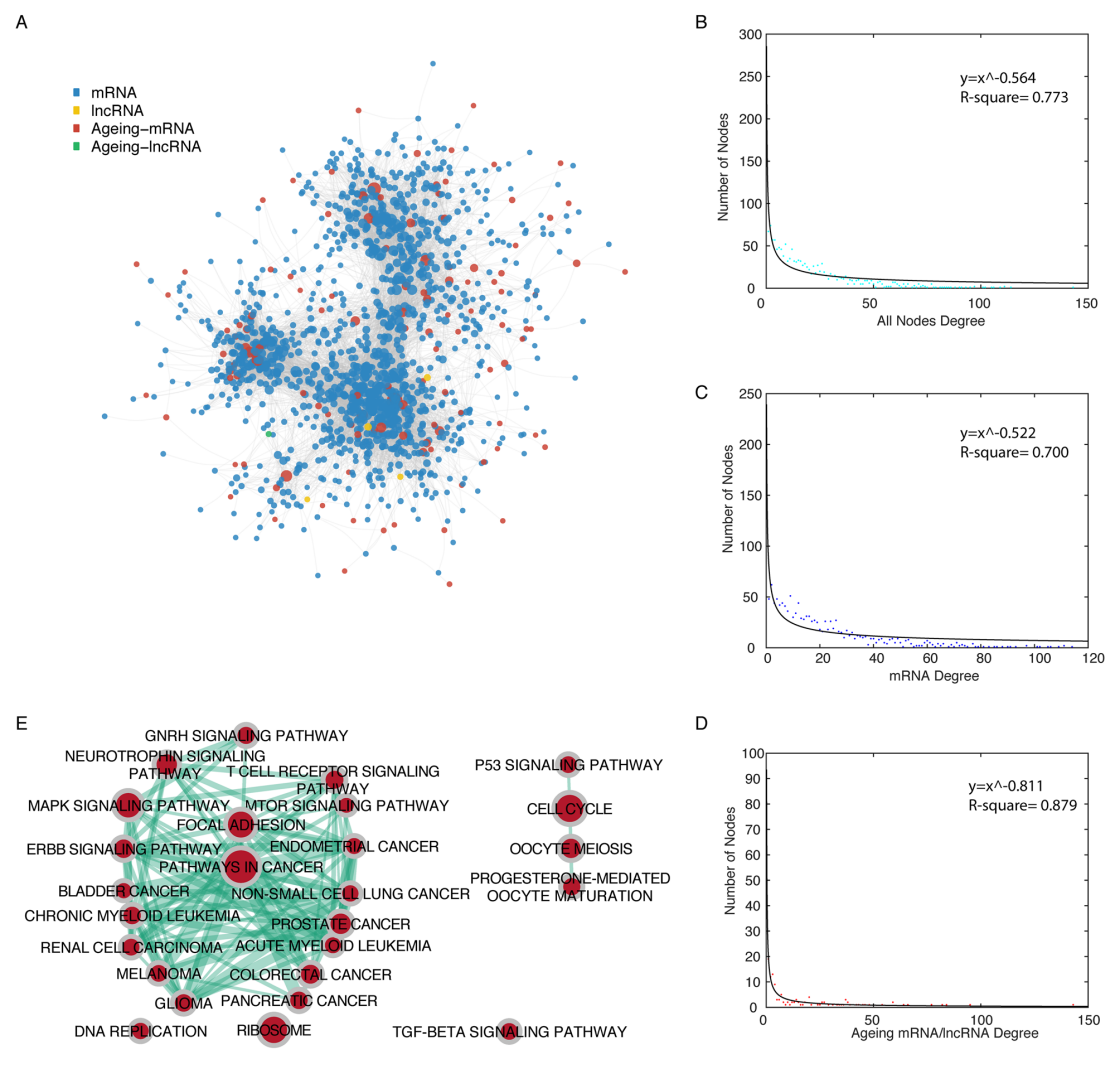
Topological properties of the ageing gene-associated ceRNA network (AgeingCeNet) **(A)** The overview of AgeingCeNet. **(B-D)** The degree distribution of all nodes, mRNA, and ageing genes in AgeingCeNet. Node colour was set according to the node colour in AgeingCeNet. **(E)** The enrichment map of KEGG pathways that enriched by genes in AgeingCeNet. Node size represents the number of genes in specific KEGG pathway. The edge thickness represents the number of genes shared by the two KEGG pathway linked by the edge.

We analysed mRNA/lncRNA expression data from 251 bladder cancer samples, along with experimentally validated miRNA-mRNA interaction data and miRNA-lncRNA interaction data to construct a functional miRNA mediated ceRNA network in bladder cancer. In total, 32415 miRNA mediated ceRNA interactions were identified, including 34 lncRNA-lncRNA, 193 lncRNA-mRNA, and 32188 mRNA-mRNA ceRNA pairs, which was used to build a bladder cancer specific ceRNA network. We then extracted an ageing gene associated ceRNA network, AgeingCeNet, which includes 1322 nodes (4 lncRNAs, 1 ageing lncRNA; 1197 mRNAs, 120 ageing mRNAs) and 13563 ceRNA pairs (82 lncRNA-mRNA ceRNA pairs, 13481 mRNA-mRNA ceRNA pairs. Figure 1A, Supplementary Table 1). We validated the correlation between the ageing genes and their ceRNAs using an independent dataset GSE87304, which contains 305 bladder cancer samples. About 95 percent of correlations between ageing genes and their ceRNAs that have expression data in this dataset were verified (p value < 0.05. Supplementary Table 2). In order to study the topological properties of AgeingCeNet, degree distribution analysis was performed for all nodes, mRNAs, and ageing genes (Figure 1B-1D). The degree distribution analysis reveals that all nodes, either ageing genes or their ceRNAs in AgeingCeNet all follow a power law distribution, which indicates that a majority of nodes in AgeingCeNet have few interactions with other nodes in AgeingCeNet, while a minority of nodes have large numbers of interactions with others.

We also performed functional enrichment analysis for all genes in AgeingCeNet based on Gene Ontology biological process terms and Kyoto Encyclopaedia of Genes and Genomes (KEGG) pathways [11]. AgeingCeNet was enriched in 338 GO biological terms and 31 KEGG pathways (p-vaule cutoff = 0.01, Supplementary Table 3). The top 20 enriched GO biological terms and KEGG pathways were visualized (Supplementary Figure 1A-1B). We also visualized and clustered the enriched KEGG pathways using EnrichmentMap (Figure 1E). Of these, 12 were cancer pathways including bladder and renal cell cancer and 9 were cancer signaling pathways including P53 [12], mTOR [13], and MAPK [14]. The GO term and KEGG pathway enrichment analyses revealed that genes in AgeingCeNet were closely associated with a variety of cancers including bladder cancer.

### The roles of ageing genes (mRNA/lncRNA), ceRNAs and hub nodes in AgeingCeNet

**Figure 2:**
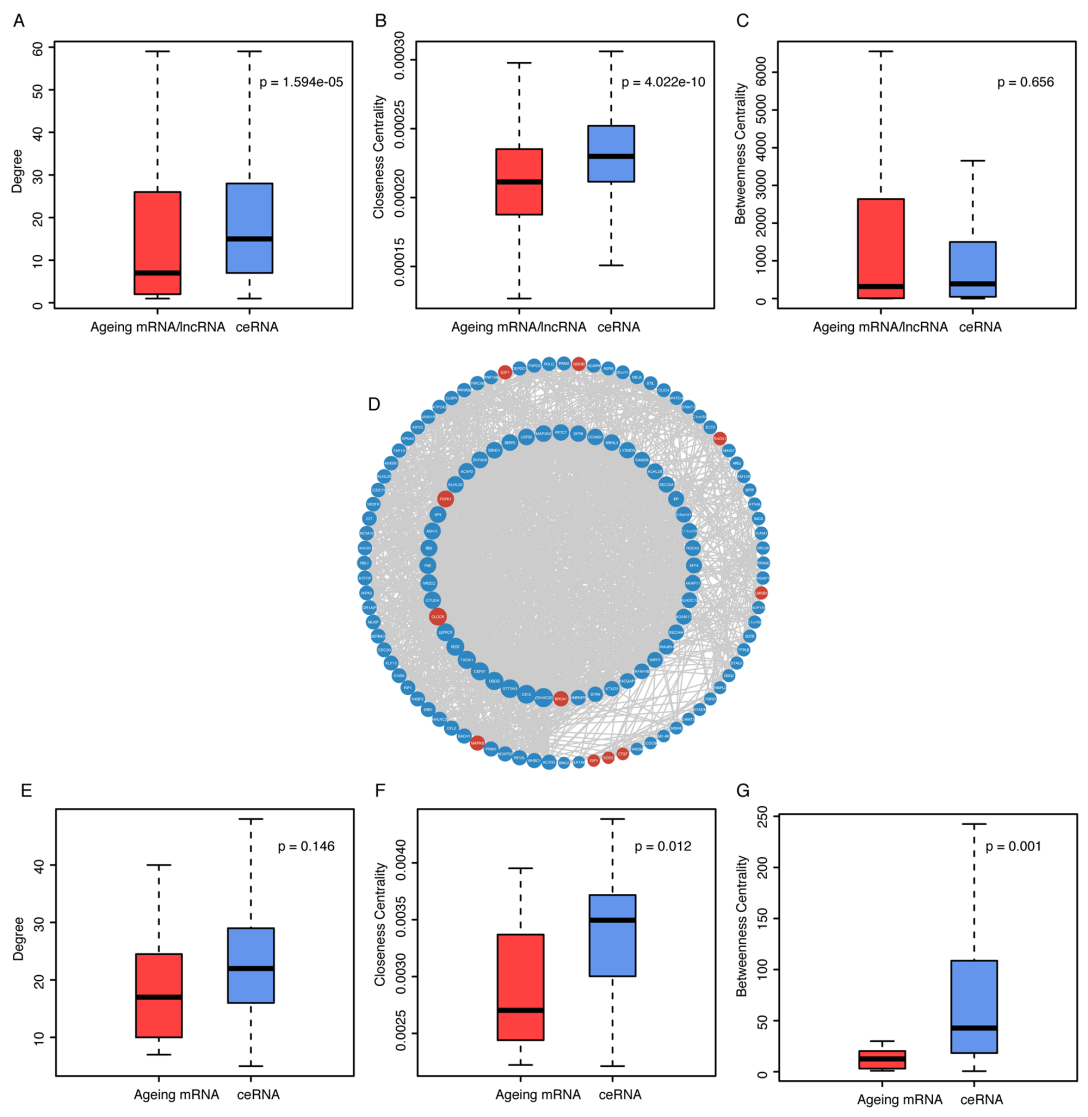
Comparison analysis of topological properties between ageing genes and their ceRNAs in AgeingCeNet and hubNet of AgeingCeNet **(A-C)** Boxplot of degree, closeness centrality and betweenness centrality of ageing genes and their ceRNAs in AgeingCeNet. **(D)** The hub network (hubNet) of AgeingCeNet. **(E-G)** Boxplot of degree, closeness centrality and betweenness centrality of ageing genes and their ceRNAs in hubNet.

We found that ceRNAs of ageing genes have a significantly higher degree than ageing genes themselves in AgeingCeNet (p-value = 1.594e-05, Figure 2A), which indicates ceRNAs of ageing genes have more interactions with other nodes than ageing genes in AgeingCeNet. Closeness centralities (CC) of ceRNAs of ageing genes are also significantly larger than that of ageing genes (p-value = 4.022e-10, Figure 2B), which reveals that ceRNAs of ageing genes have smaller average length of the shortest paths to all the other nodes than ageing genes in AgeingCeNet. Notably, the betweenness centrality difference between ageing genes (Figure 2C) and their ceRNAs is not significant (p-value = 0.656), indicating that they may have a similar communication function in AgeingCeNet.

For further analysis, we took the hub nodes defined as the top 10% highest degree nodes of AgeingCeNet to construct a hub network (hubNet). The network contains 132 nodes (11 ageing mRNAs and 121 ageing gene ceRNAs) and 1489 edges (Figure 2D). Although the difference of degree centrality between ageing mRNAs and their ceRNAs is not significant in hubNet (p-value = 0.146, Figure 2E). The closeness and betweenness centrality of ageing mRNAs ceRNAs are significantly larger than that of ageing mRNAs themselves (p-value = 0.012 and 0.001, respectively, Figure 2F, 2G), suggesting that these ageing mRNAs associated ceRNAs function in a crucial communication role in hubNet of AgeingCeNet.

There were several verified cancer-related ageing mRNAs in the hubNet of AgeingCeNet. CLOCK gene (Ageing gene) as the node with the highest degree in AgeingCeNet was verified to be associated with urothelial cancer [15]. BRCA1, another hub ageing gene, is associated with breast, ovarian, prostate, pancreas and stomach cancer [16]. MAPK8, MAP kinase family, is associated with cell proliferation, apoptosis and differentiation and was confirmed to be associated with a variety of cancers [17].

### Identification of prognostic module biomarkers from AgeingCeNet in bladder cancer

**Figure 3:**
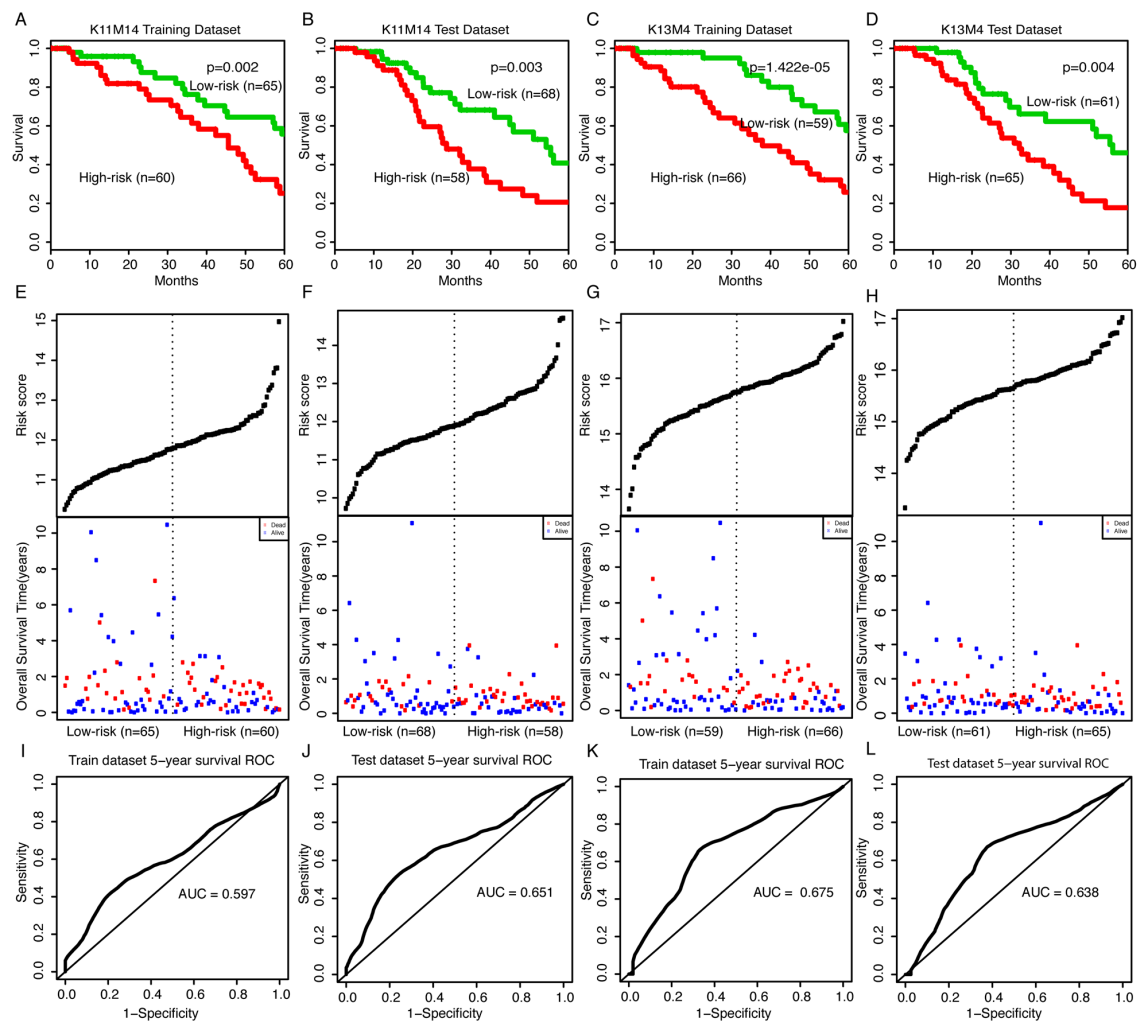
Survival curves and risk score analysis of module K11M14 and K13M4 in training and test dataset **(A-D)** Kaplan-Meier survival curve for overall survival of training and test data set with high and low risk score of K11M14 and K13M4, respectively. **(E-H)** Risk score analysis of K11M14 and K13M4 for training and test dataset. **(I-L)** Receiver operating characteristic (ROC) curve analysis and area under the curve (AUC) value of the ROC curve indicating the sensitivity and specificity of K11M14 and K13M4 for survival prediction in training and test dataset, respectively.

We obtained 37 modules from AgeingCeNet using CFinder software with threshold K-clique > 10 (Supplementary Table 4). For each module, we used multivariate Cox regression model with overall survival time as the dependent variable and module genes as covariates. A risk score for each module gene was measured by the regression coefficient derived from the multivariate Cox regression analysis (Supplementary Table 5). The risk score for each module was obtained by a linear combination of module gene expression value weighted by the gene risk score (regression coefficient mentioned above). Two hundred-fifty one (251) bladder samples having mRNA/lncRNA expression and clinical data were randomly divided into two subsets [18]: a training set (125 samples) and a test set (126 samples). For each module, we used the average module risk score of the training set samples to divide both the training set and test set into two subsets: high risk score samples and low risk score samples. We identified two modules that had significant prognostic ability both in training and test set (Supplementary Table 6). One module, comprised of 12 genes, was the fourteenth module derived from CFinder with K-clique = 11, named K11M14. For this module, the numbers of high risk samples were 60 in the training dataset and 58 in the test dataset, and the numbers of low risk samples were 65 in the training dataset and 68 in the test dataset. The high risk score samples had a significantly shorter survival time than the low risk score samples both in the training set and the test set (log rank p-value = 0.002 and 0.003, respectively, Figure 3A, 3B). The other module that included 16 genes (15 mRNAs and 1 lncRNA) is the fourth module derived from CFinder with K-clique = 13, named K13M4. For this module, the numbers of high risk samples were 66 in the training dataset and 65 in the test dataset, while the numbers of low risk samples were 59 in the training dataset and 61 in the test dataset. The high risk score samples had significantly shorter survival time than the low risk score samples both in the training set and the test set (log rank p-value = 1.442e-05 and 0.004, respectively, Figure 3C, 3D). The distribution of gene risk scores and the survival statuses for the two modules in training dataset and test dataset are shown in Figure 3E-3H. Patients with high-risk scores tended to present poorer clinical outcomes compared to patients with low-risk scores. To evaluate the sensitivity and specificity of the survival prediction of the two modules, we adopted a time-dependent receiver operating characteristic (ROC) curve analysis for training dataset and test dataset. The values of area under the curve (AUC) for K11M14 module were 0.597 and 0.651 in the training and test dataset, respectively (Figure 3I, 3J). The values of area under the curve (AUC) for K13M4 module were 0.675 and 0.638 in the training and test dataset, respectively (Figure 3K, 3L). These results indicate that the two modules both have a superior prediction performance.

Cancer statistics data projected that 143,190 new cases were to be diagnosed (58,950 in male and 18,010 in female) and 31,540 deaths were to be reported (11,820 in male and 4,570 in female) in 2016 [19]. The number of newly diagnosed or deaths from bladder cancer in male is twice of that in female, which suggests that gender should be a factor for bladder cancer. Age is also a risk factor for bladder cancer. Therefore, univariate Cox regression was performed for module risk score, gender, and age for each module in the training and test dataset. We also performed another multivariate Cox regression model with overall survival time as the dependent variable and module risk score, age and gender as covariates for K11M14 and K13M4 in the training set and test set. The results are shown as Table 1 and demonstrate that K11M14 and K13M4 both have more significant prognostic ability than gender and age in each data set.

**Table 1:** Univariate and multivariate Cox regression analysis of module K11M14 and K13M4 in bladder cancer patients

	Variables	Univariate analysis			Multivariate analysis		
		HR	95% CI of HR	P-value	HR	95% CI of HR	P-value
**K11M14 training dataset**	Risk Score	2.718	1.680-4.398	4.63E-05	2.701	1.692-4.309	3.09E-05
	Gender	0.668	0.369-1.212	0.184	0.641	0.353-1.164	0.144
	Age	1.018	0.989-1.047	0.229	1.018	0.989-1.048	0.225
**K11M14 test dataset**	Risk Score	1.632	1.213-2.196	0.001	1.689	1.233-2.313	0.001
	Gender	1.538	0.768-3.080	0.225	1.840	0.895-3.784	0.097
	Age	1.013	0.984-1.043	0.374	1.009	0.980-1.039	0.538
**K13M4 training dataset**	Risk Score	2.718	1.514-4.881	0.001	2.761	1.522-5.009	0.001
	Gender	0.668	0.369-1.212	0.184	0.799	0.436-1.466	0.469
	Age	1.018	0.989-1.047	0.229	1.022	0.992-1.053	0.150
**K13M4 test dataset**	Risk Score	1.873	1.182-2.969	0.008	1.884	1.177-3.015	0.008
	Gender	1.538	0.768-3.080	0.225	1.393	0.688-2.820	0.357
	Age	1.013	0.984-1.043	0.374	1.020	0.990-1.052	0.190

Note: HR represents hazard ratio, CI represents confidence interval.

### miRNAs that regulate prognostic module

**Figure 4:**
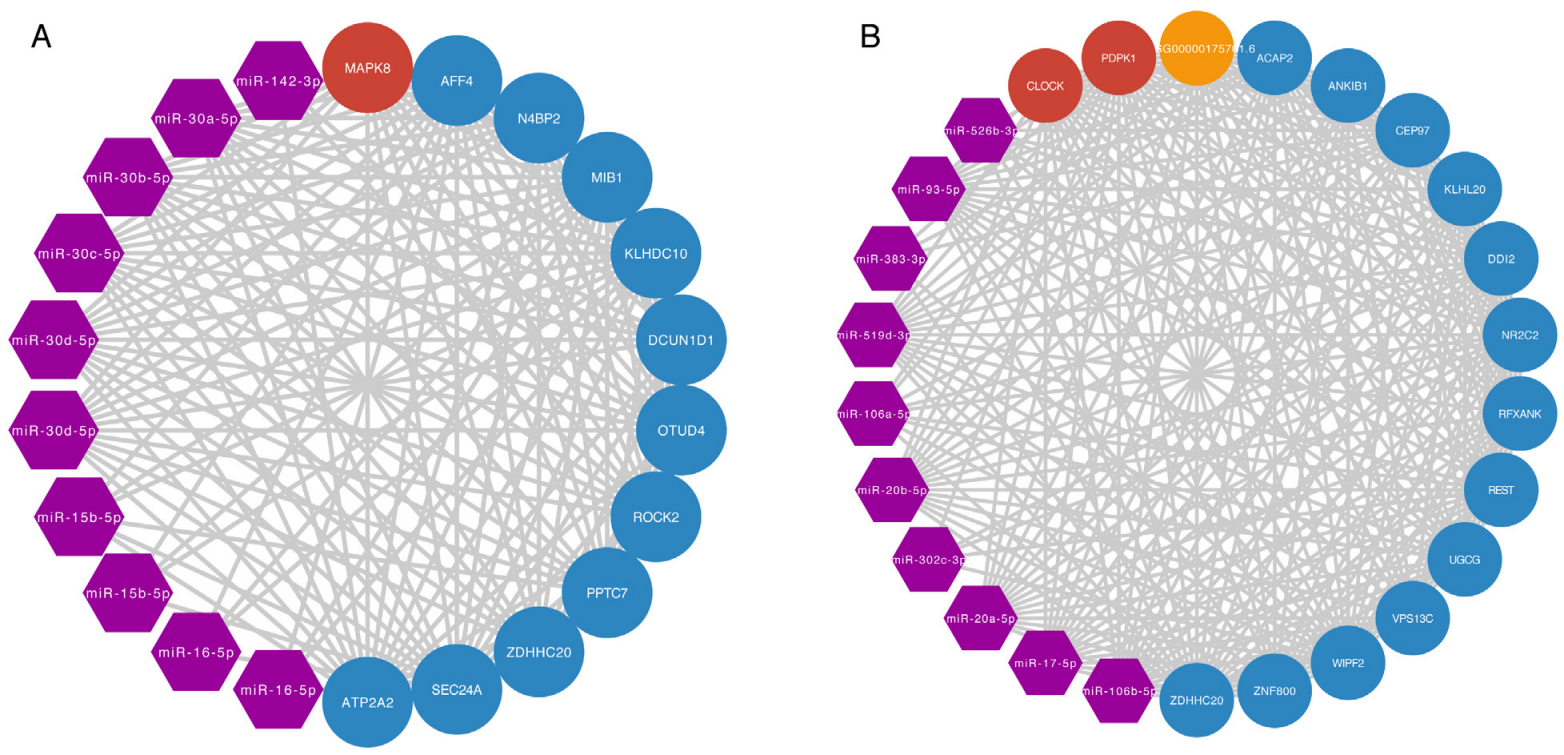
miRNA regulation of prognostic modules network **(A, B)** miRNA regulation of prognostic modules network for K11M14 and K13M4, respectively. The purple, red, orange, and blue nodes represent miRNA, ageing mRNA, ageing lncRNA and ageing gene associated ceRNA, respectively.

To explore which miRNAs play crucial roles in regulation of the two prognostic modules. We listed the miRNAs that were shared by ceRNA pairs in the two modules (Supplementary Table 7). We found the top 10 miRNAs that were shared by ceRNA pairs in the two modules, respectively (Figure 4A, 4B). Notably, five miRNAs of top 10 K11M14 regulating miRNAs belong to miR-30 family, which can regulate the growth of cancer cells [20]. miR-142-3p was shown to be a tumour suppressor for several cancers [21, 22]. miR-15b-5p was identified as a potential urine biomarker for bladder cancer [23]. miR-195-5p was shown to suppress cell proliferation in bladder cancer [24]. K13M4 regulating miRNAs miR-17-5p and miR-20a were found previously to be overexpressed in bladder cancer [25]. In summary, our method not only identified two prognostic modules, but also at least 3 potential miRNA biomarkers for bladder cancer.

## DISCUSSION

There is considerable urgency to identify novel therapeutic targets to improve the diagnosis, prognosis prediction, and ultimately survival outcomes in bladder cancer. Considering age is one of the most important risk factors for cancer and a ceRNA mechanism has been verified to play an important role in cancer biology, we constructed an ageing gene related ceRNA network (AgeingCeNet) specific to bladder cancer. KEGG Enrichment analysis for AgeingCeNet genes indicated that these genes were enriched in specific types of signaling and cancer pathways (including bladder cancer). Network analysis results implied that ceRNAs of ageing genes have significantly more interactions than ageing genes in AgeingCeNet. Moreover, ceRNAs of ageing genes tend to be in the centre of AgeingCeNet and play a similar communication role with ageing genes indicating both play important roles in the network.

Furthermore, we identified two potential prognostic modules (K11M14 and K13M4) in AgeingCeNet. Both modules had a better prognostic ability than age and gender which are considered as common risk factors for bladder cancer [26]. The two modules contain several validated cancer-associated genes. MAPK8, the only ageing gene in K11M14, is a member of the MAP kinase family, which play roles in cell proliferation, differentiation and other cellular processes. It’s also an essential member of the MAPK signaling pathway, which is a common mutational activation signaling pathway in bladder cancer [27]. The MAPK8 ceRNAs in K11M14 can regulate the expression of MAPK8 by competing shared miRNAs, which might be potential biomarkers for bladder cancer. PDPK1, an ageing gene in K13M4, is a master kinase, which can function downstream of PI3K through PDPK1’s interaction with membrane phospholipids. PI3K pathway is another mutational activation signaling pathway in bladder cancer [27]. The ceRNAs of PDPK1 in K13M4 might be potential biomarkers for bladder cancer. Notably, the only lncRNA ENSG00000175701.6 (also known as LINC00116), one ceRNA of PDPK1, in K13M4 was identified as a potential biomarker for lung cancer [28]. By constructing an ageing gene related ceRNA network for bladder cancer, we identified two prognostic modules that have a close relationship with two common mutational activation signaling pathways, which suggests that exploring the mechanism from the perspective of ageing might be a feasible strategy.

In conclusion, by constructing an ageing gene related ceRNA network, we identified two prognostic modules and related regulating miRNAs in bladder cancer, which both contain several cancer related molecules. Ageing gene ceRNA network construction and analysis was shown as a feasible strategy to explore the mechanism of bladder cancer and might benefit its diagnosis and treatment.

## MATERIALS AND METHODS

### Workflow

**Figure 5:**
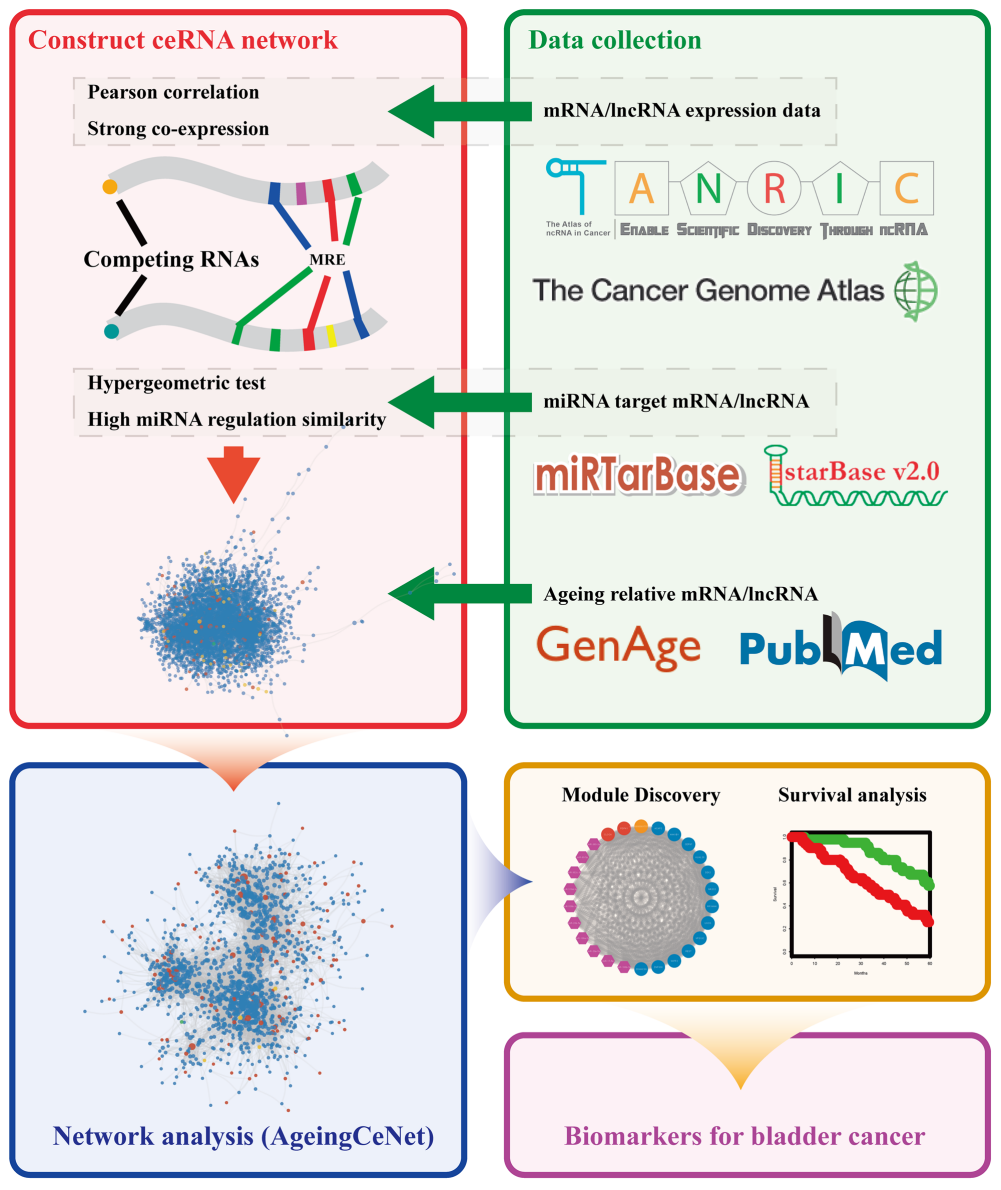
Workflow of the study Different colour frames represent processes in this study, including data collection, network construction, module discovery, survival analysis and biomarker discovery.

The workflow is shown in Figure 5 and includes mRNA/lncRNA expression data, miRNA targets data and ageing associated mRNA/lncRNA collection, bladder cancer specific ceRNA construction, AgeingCeNet construction, network analysis, identification of prognostic modules and potential biomarkers for bladder cancer.

### Data collection

The RNASeq V2 level 3 data and clinical data for bladder cancer were downloaded from The Cancer Genome Atlas (TCGA). We obtained 427 samples with gene expression data based on the “gene_exp_rpkm” value and 412 samples with clinical data. We removed 4 samples from clinical samples with negative overall survival time. To filter genes that were not expressed across most samples in RNA-seq datasets, we removed genes with expression value = 0 in more than 50% of samples. The gene expression values were log2 transformed. The lncRNA expression data of 271 samples were downloaded from TANRIC, which is an interactive open platform to explore the function of lncRNAs in cancer. TANRIC used the dataset from TCGA. Finally, we obtained 251 overlapping samples with gene expression data, lncRNA expression data and clinical data.

Another mRNA expression dataset GSE87304 on the Affymetrix HuEx-1_0-st platform was downloaded from Gene Expression Omnibus (GEO) database [29]. The dataset contains 305 bladder cancer samples and was used to validate the correlation between aging genes and their ceRNAs.

Experimentally verified human miRNAs and targets were obtained from miRTarBase (version 6.1) [30]. A total of 324,219 non-redundant experimentally verified miRNA-target interactions were used in our analysis. The experimentally validated miRNA-lncRNA interactions were downloaded from starBase v2.0 [31] including 10,212 miRNA-lncRNA interactions.

Human ageing associated mRNAs were downloaded from The Ageing Gene Database (GenAge) [32]. In addition, ageing associated lncRNAs were extracted from a published report [33]. In total, 305 human ageing associated mRNAs and 30 ageing associated lncRNAs (Supplementary Table 8) were used as the seed nodes to construct AgeingCeNet.

### Construction of ageing genes associated ceRNA network

Our hypothesis was that ceRNA pairs should satisfy two criteria: one is the pair should have high miRNA regulation similarity. The other is that the pair should have strong co-expression [18, 34, 35]. Firstly, a hypergeometric test was used to evaluate the significance of shared miRNAs for each possible ceRNA pair. For a given RNA pair A and B, we identified the RNA pair of shared miRNAs at first. Then, we calculated the probability of sharing miRNAs for A and B as follows:

P=1−F(x|N,K,M)=1−∑t=0x−1(Kt)(N−KM−t)(NM)

Where N represents the number of all miRNAs in study. K and M represents the number of miRNAs that target RNA A and RNA B, respectively. x represents the number of miRNAs targeting both RNA A and RNA B. Only the pairs that satisfy x ≥3 and FDR-adjusted p-value < 0.01, were reserved for the following analysis. Secondly, Pearson correlation coefficient was used to evaluate the co-expression of RNA A and RNA B. The formula is shown below:

p(A,B)=cov(A,B)σ(A)σ(B)

Where *cov*(A, B) represents the covariance of gene expression values of RNA A and B. σ(A) and σ(B) represent the standard deviation for gene expression values of RNA A and B, respectively. Only the pairs that satisfy absolute Pearson correlation coefficient > 0.5 and FDR-adjusted p-value < 0.01, were considered as ceRNA candidates.

According to the two principles, a bladder cancer specific ceRNA network was constructed. Ageing genes and RNAs directly interacting with ageing genes in the ceRNA network were selected and extracted as a new subnetwork using Cytoscape 3.5.1 [36]. The maximal connected component of the subnetwork was defined as AgeingCeNet.

### Network analysis

To study the structure of AgeingCeNet, we performed topological properties analysis including degree, closeness centrality (CC) and betweenness centrality (BC). Degree of a node is the number of nodes that interact with the node. CC is defined as:

CC(v)=1∑j≠vdv,j

Where d_v, j_ represents the shortest path from node v to node j. In brief, a node with big CC is much closer to other nodes. In other words, a node with a big CC tends to be at the centre of the network. BC of a node v is measured as below:

BC(v)=∑s≠t≠vσst(v)

Where σ_st_(v) represents the number of shortest paths from node s to node t that passes node v. In brief, BC can reflect the role of a node in communication [37].

The network analysis, visualisation, and modules discovery was implemented by a R package “igraph”, Cytoscape and CFinder [38].

### Functional enrichment analysis

KEGG pathway enrichment analysis was implemented using DAVID Bioinformatics Resources version 6.7 (https://david-d.ncifcrf.gov) [39, 40]. Functional categories were visualized and clustered using the EnrichmentMap [41], a plugin in Cytoscape 3.5.1.

### Survival analysis

The risk score (RS) of a module was calculated using the formula:

RS=∑i=1nR(i)Exp(i)

Where n represents the number of module RNAs. *R*(*i*) represents the estimated regression coefficient of RNA i from the multivariate Cox regression analysis. *Exp*(*i*) is the expression value of RNA i. If the RS of a given sample is bigger than the mean of RS of all samples, then the sample is classified as a high risk sample. Otherwise, the sample is classified as a low risk sample. The Kaplan-Meier (KM) method was used to estimate the survival curves between high risk and low risk groups. The two-sided log-rank test was used to evaluate the statistical significance of the survival curves. Additionally, receiver operating characteristic (ROC) curve analysis and the area under the ROC curve (AUC) were used to evaluate the sensitivity and specificity of survival prediction.

## SUPPLEMENTARY MATERIALS FIGURE AND TABLES

















## Abbreviations

AgeingCeNet: an ageing gene related ceRNA network
AUC: area under the curve
BC: betweenness centrality
CC: closeness centrality
ceRNA: Competitive endogenous RNA
KEGG: Kyoto Encyclopedia of Genes and Genomes
lncRNA: long non-coding RNA
miRNA: microRNA
MRE: miRNA response elements
mRNA: messenger RNA
ROC: receiver operating characteristic
TANRIC: The Atlas of non-coding RNA in Cancer
TCGA: The Cancer Genome Atlas
