# Supplementary material for: Identification of bladder cancer prognostic biomarkers using an ageing gene-related competitive endogenous RNA network
Source: Oncotarget. 2017 Dec 4;8(67):111742–53. doi: 10.18632/oncotarget.22905 (PMC5762356; doi:10.18632/oncotarget.22905)
Supplement: Supplementary file 1 [file oncotarget-08-111742-s001.pdf]

Identification of bladder cancer prognostic biomarkers using an ageing gene-related competitive endogenous RNA network

SUPPLEMENTARY MATERIALS

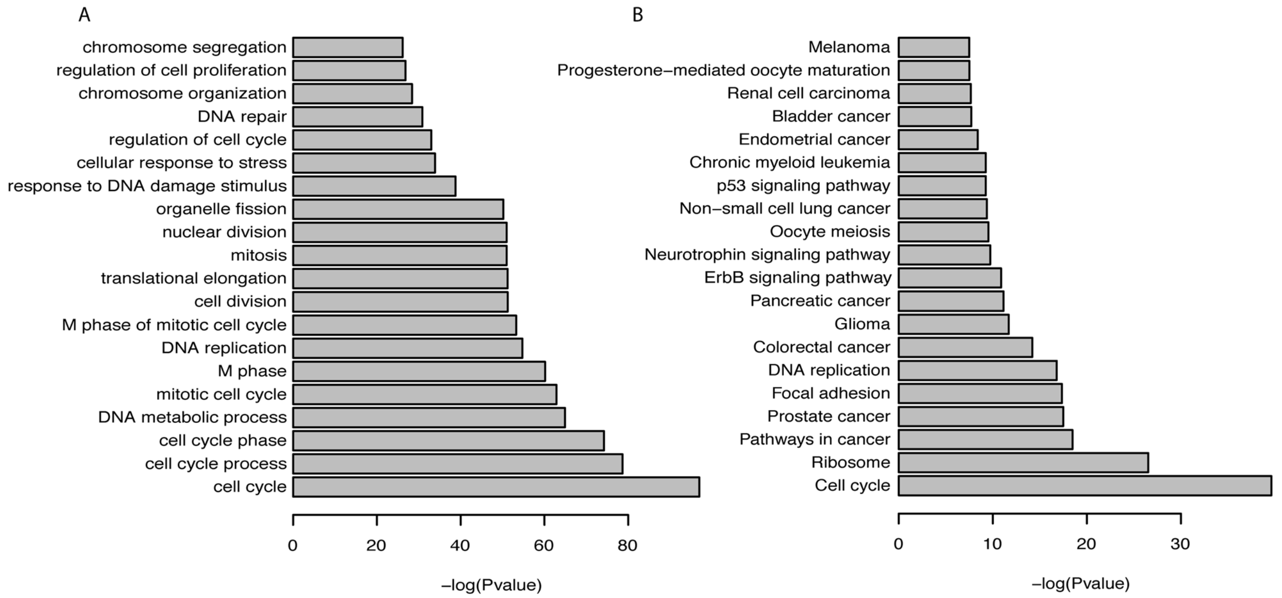

Supplementary Figure 1: GO and KEGG enrichment analyses of AgeingCeNet genes. (A) GO enrichment analysis of AgeingCeNet genes. The x-axis shows the value of negative log transformed of p value. The y-axis shows the top 20 enriched GO biological terms. **(B)** KEGG enrichment analysis of AgeingCeNet genes. The x-axis shows the value of negative log transformed of p value. The y-axis shows the top 20 enriched KEGG pathways.

Supplementary Table 1: Interactions in AgeingCeNet

See Supplementary File 1

Supplementary Table 2: Ageing genes and their ceRNAs validation results

See Supplementary File 2

Supplementary Table 3: Enriched KEGG pathways of mRNAs in AgeingCeNet

See Supplementary File 3

Supplementary Table 4: Cox regression coefficient table for each gene of modules identified by Cfinder

See Supplementary File 4

Supplementary Table 5: The log rank p-values and mean risk scores summary table for modules identified by CFinder

See Supplementary File 5

Supplementary Table 6: K11M14 and K13M4 ceRNA pairs and related miRNA list

See Supplementary File 6

Supplementary Table 7: Ageing mRNA and ageing lncRNA list

See Supplementary File 7
